# SIMPLIFIED LAPAROSCOPIC CHOLECYSTECTOMY WITH TWO INCISIONS

**DOI:** 10.1590/S0102-67202014000200014

**Published:** 2014

**Authors:** Rafael Antoniazzi ABAID, Ivan CECCONELLO, Bruno ZILBERSTEIN

**Affiliations:** 1 Hospital Santa Cruz, UNISC (Santa Cruz Hospital, UNISC), Santa Cruz do Sul, RS, Brazil.; 2 Hospital das Clínicas, Faculdade de Medicina, Universidade de São Paulo (Clinic Hospital, School of Medicine, University of São Paulo), São Paulo, SP, Brazil.

**Keywords:** Cholecystectomy, laparoscopic, Cholecystitis, Cholelithiasis, Minimally invasive surgical procedures

## Abstract

**Background:**

Laparoscopic cholecystectomy has traditionally been performed with four incisions to insert four trocars, in a simple, efficient and safe way.

**Aim:**

To describe a simplified technique of laparoscopic cholecystectomy with two incisions, using basic conventional instrumental.

**Technique:**

In one incision in the umbilicus are applied two trocars and in epigastrium one more. The use of two trocars on the same incision, working in "x" does not hinder the procedure and does not require special instruments.

**Conclusion:**

Simplified laparoscopic cholecystectomy with two incisions is feasible and easy to perform, allowing to operate with ergonomy and safety, with good cosmetic result.

## Abstract (pt)

**Racional:**

A colecistectomia videolaparoscópica vem tradicionalmente sendo realizada através de quatro incisões para inserção de quatro portais, de maneira simples, eficiente e segura.

**Objetivo:**

Descrever técnica de colecistectomia videolaparoscópica simplificada com duas incisões, utilizando instrumental convencional básico.

**Técnica:**

Na incisão da cicatriz umbilical aplicam-se dois portais e no epigástrio mais um portal. O uso de dois portais na mesma incisão, trabalhando em “x”, não dificulta a realização do procedimento e não requer instrumental especial.

**Conclusão:**

A colecistectomia videolaparoscópica simplificada com duas incisões é técnica de fácil execução, permitindo operar com ergonomia, segurança e com bom resultado estético.

## INTRODUCTION

The evolution of laparoscopic surgery still faces many challenges. One is to become even less invasive. Laparoscopic cholecystectomy has traditionally been performed with four portals, simple, efficient and at low cost. Other ways have been described. To reduce the number of portals and achieve better cosmetic results, the authors have used wire traction in the gallbladder in place to forceps^9,11^. Also, is described the use optical channels like work way^15^. Meanwhile, the operation through natural orifices (NOTES) has been used only in protocols^7,13^. Although the new procedures try to reduce the number of portals and incisions, they increase the technical difficulties, the risk of complications and costs, which has been a barrier to its implementation. Of these procedures, the two most used are: minilaparoscopia^10,14,4,16,7,8,9^ and umbilical cholecystectomy through a single incision^2,6,3,5,17,8,16,12,18^.

Cholecystectomy by minilaparoscopy is very similar to conventional laparoscopic cholecystectomy, but uses smaller diameter trocar with delicate tweezers^14^. Thus, there is greater wear and shorter life of the device, increasing the cost. However, it has the advantage of using devices similar to conventional technique, which needs no further training. However, it requires four incisions, with an umbilical incision of 10 mm for the use of optics and removal of gallbladder^14,1^ and requires skill to do intracorporeal knot ligation of the cystic duct .

In single-incision cholecystectomy the procedure is performed by only one transumbilical incision; however, the incision is usually measured approximately 3 cm, beyond the limits of the umbilicus. It is preferably carried out with the use of a single portal and curved special clamps, which increases the cost^5,17^. It may be accomplished with conventional instruments, but with lower angle between the clamps. Presents greater technical difficulty and longer learning curve^3^. As risk, may have even higher incidence of incisional hernia. Often this technique is used with a secondary incision in the right flank or right hypochondrium to better expose the operative field with additional traction^5^, which takes away the advantage of the single portal.

With the aim of reducing the number of incisions without using special materials and without increasing the technical difficulty, the authors propose a hybrid simplified laparoscopic technique for cholecystectomy with two incisions.

## TECHNIC

**Figure 1:**
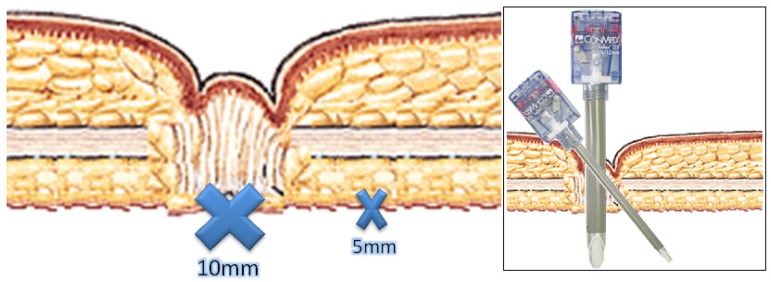
Positioning of the two trocars in single umbilical incision

The procedure is performed under general anesthesia and the patient supine in slight inclination positon. Incision is held within the umbilicus about 15 mm. After completion of the pneumoperitoneum, abdominal incision is made for a 10 mm trocar. A second 10 mm trocar is inserted below the xiphoid process. With 30º 10 mm optical device in the epigastric portal, is possible to have vision of the insertion of a second 5 mm trocar inside the umbilical incision next to the 10 mm already inserted, penetrating the aponeurosis laterally to it (Figure 1).

**Figure 2:**
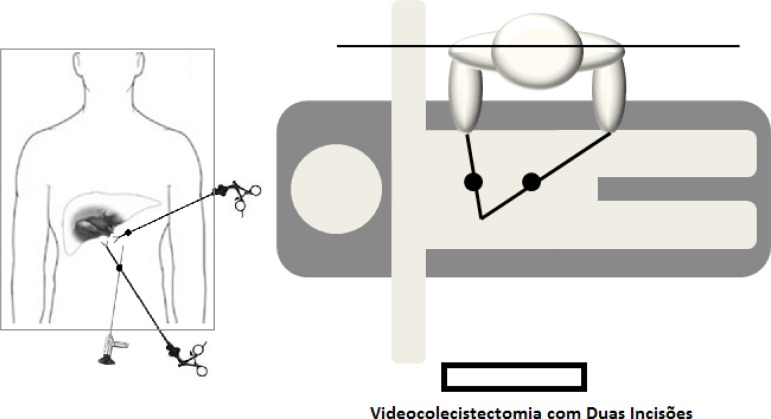
Positioning of the instruments of labor and its ergonomic manipulation by the surgeon

The procedure begins with the optics on the umbilicus, gripping forceps on the portal of 5 mm and a Maryland forceps in epigastric incision (Figure 2).

**Figure 3:**
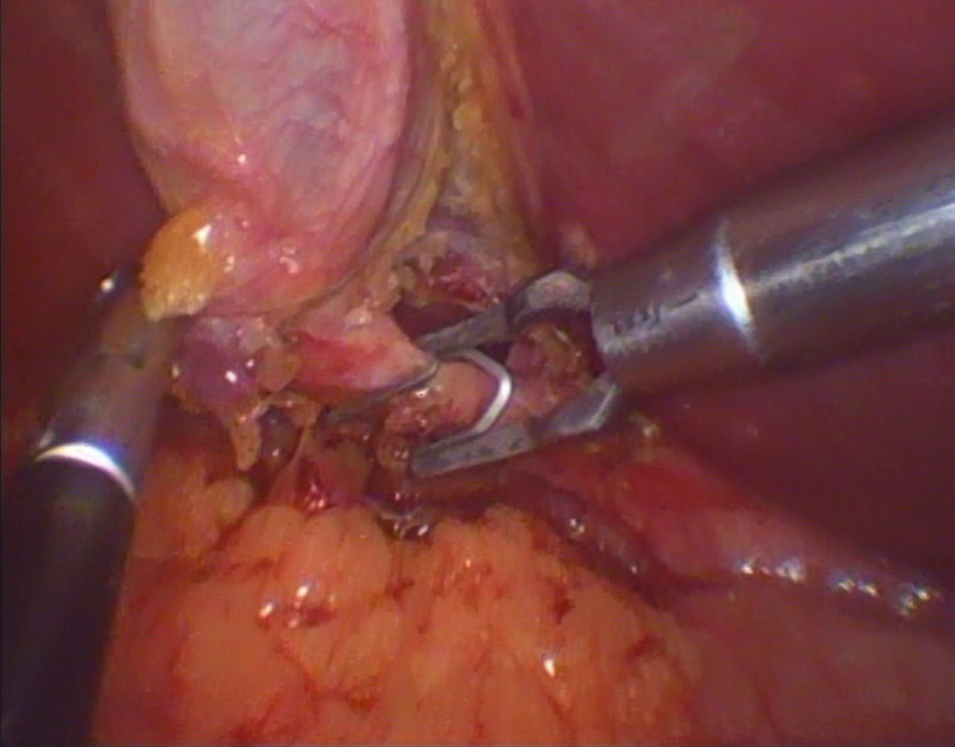
Placement of clips closing the cystic duct

**Figure 4:**
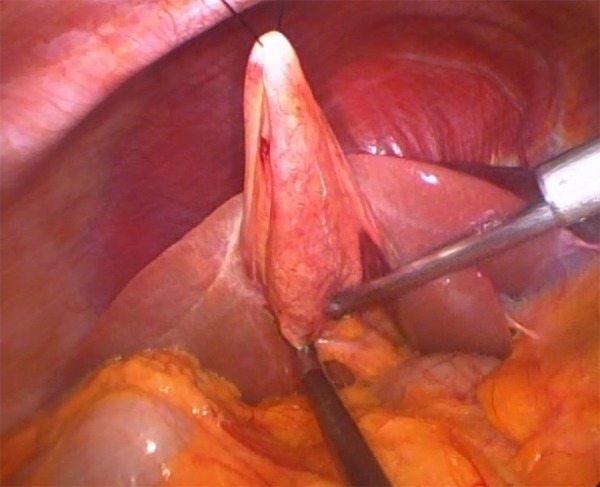
Dissection of the gallbladder with electrocautery

A mononylon 000 with straight needle is inserted through the abdominal wall just below the right costal margin in the right midclavicular line. It transfix the body wall of the gallbladder and the needle is externalized near the site of entry into the cavity, rising and pulling the gallblader, exposing the cisto-hepatic triangle (Calot, Figures 3 and 4).

**Figure 5:**
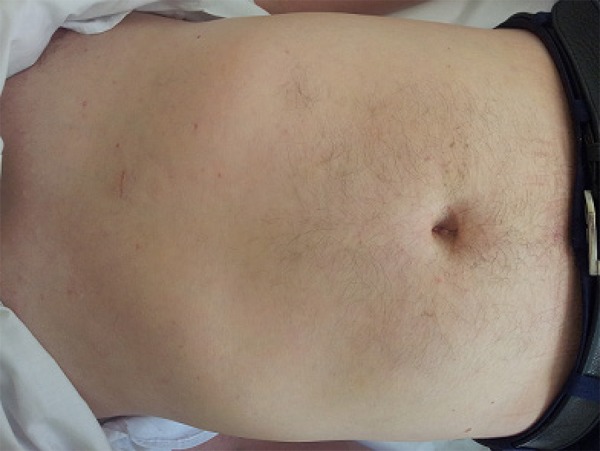
Insertion of the catheter in the cystic duct cholangiography and final aspect of the operation after 60 days

The gripper is used to grip vesicular infundibulum and the trigone dissection is performed in the usual manner, through the epigastric portal. The cystic duct and artery are ligated with metal clips (Figure 3). Intraoperative cholangiography is conducted through intracath type 14G by transfixion of the abdominal wall (Figure 5). Then the gallbladder is dissected from the liver bed with hook electrocautery. The wire pulling the gallblader is removed only at the moment when it is placed inside the extractor bag and withdrawn through the hole created for the epigastric portal.

## RESULTS

This procedure was applied to 10 sequential patients, one male and nine female, mean age 38 years (21-62), with a mean operative time of 66 minutes (42-88) without complications. In three patients the clinical findings were of acute cholecystitis with intervention in emergency. In seven the procedure was elective. All patients were discharged within 24 hours.

## DISCUSSION

The procedure uses only basic conventional material. No ancillary puncture is used. The first trocar insertion is performed according to the conventional technique, as used in any laparoscopic operation. The two subsequent punctures are made with direct visualization. The wire traction is applied on the body of the gallbladder, near the infundibulum, to achieve higher elevation next to liver. The handling of vesicular infundibulum is accomplished through the trocar inserted 5 mm from the umbilicus. Due to not be much need to move this instrument, there is little impact on umbilical portal instruments. In addition, 30º optics is used medially, while the clamp pulls inferoanterolaterally the infundibulum. Thus, the portals work in "X" manner, allowing adequate freedom of movement of the instruments (Figure 2). The dissection is performed with complete freedom by the right hand of the surgeon, just as in conventional laparoscopic technique. Thus, dissection and ligation of the cystic duct and artery are performed in the conventional manner (Figure 3) with two clamps working at an angle of 90°. Thus, the surgeon is also free to insert the cholangiography catheter in the cystic duct (Figure 5). The gallbladder is dissected from the liver bed easily, but at the end of the dissection can be lower traction on the vesicle.

It should be mentioned that some difficulties may occur, such as bile leakage due to the use of thread traction in the gallbladder, the draw of the gallblader at the end of its detachment, loss of gas in the collision between the umbilical portals. Routinely, the gallbladder is made empty at the beginning of the procedure, minimizing the leakage of bile. When there is a collision, the simple repositioning of portals solves the problem, i.e, the optic changes positions with tweezers posteriorly and vice-versa.

When compared to the technique using a single incision, it uses the same concept of reducing the incisions; however, the use of only two instruments in the umbilicus greatly reduces the incidence of collision of the device, allowing greater freedom of movement. The main difference is the use of a second incision to the working instrument of the surgeon that determines perfect triangulation between the clamps, allowing safe dissection in a similar manner to the conventional technique. No special equipment is necessary, even special abilities. It should also be noted that it is common in the art of using a single incision cholecystectomy, who use ancillary material such as endoloop or auxiliary tweezers in the right upper quadrant, making the hybrid technique and therefore eventually add more punches and thereby decreasing the possible aesthetic advantages of this procedure.

From the aesthetic point of view, this technique is superior to the conventional one, since only involves two scars (umbilical and epigastric) with the advantage of avoiding two incisions: one in subcostal site and another on the right (Figure 5).

**Figure 6:**
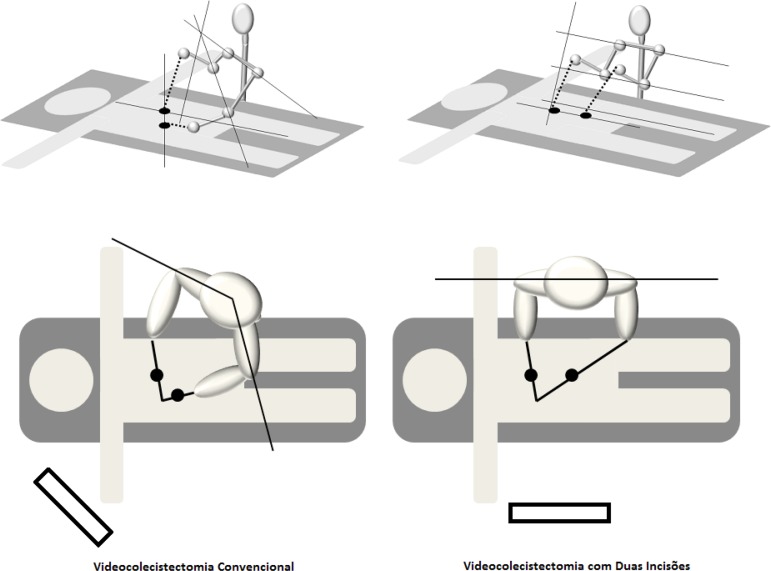
Comparison of positioning and handling of surgical instruments in laparoscopic operation with four and two incisions, demonstrating its application on ergonomic and comfortable way

The use of the traction instrument over gallblader infundibulum in umbilicus allows the surgeon to work with shoulders and elbows in straight position; so, in more ergonomic way than the conventional technique (Figure 6).

## CONCLUSION

A simplified technique of laparoscopic cholecystectomy with two incisions is feasible, safe and with superior cosmetic results compared to conventional cholecystectomy.
